# Risk-Stratified Association of Lipoprotein(a) With Carotid Plaque Burden in Middle-Aged Adults

**DOI:** 10.1016/j.jacadv.2026.103038

**Published:** 2026-07-22

**Authors:** Anastasios Kollias, Dimitra Krystallaki, Aikaterini Komnianou, Thomas Tsaganos, Emelina Stambolliu, Eirini-Chrysovalanti Antonogiannaki, Konstantinos G. Kyriakoulis, George S. Stergiou

**Affiliations:** Hypertension Center STRIDE-7, National and Kapodistrian University of Athens, School of Medicine, Third Department of Medicine, Sotiria Hospital, Athens, Greece

**Keywords:** carotid atherosclerosis, cardiovascular risk, lipoprotein(a), primary prevention

**Figure:**
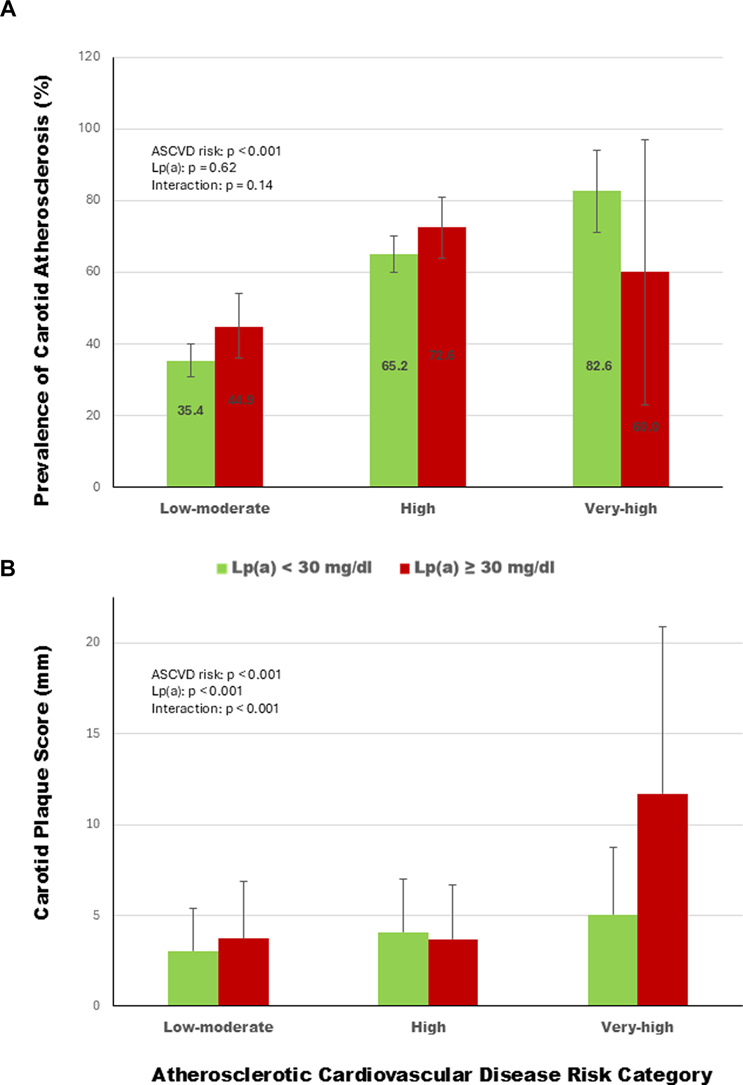

**What is the clinical question being addressed?**

Does lipoprotein(a) relate to the presence and burden of subclinical carotid atherosclerosis?

**What is the main finding?**

Lipoprotein(a) was not associated with plaque presence, but was associated with greater plaque burden, particularly at very-high atherosclerotic cardiovascular risk.

Elevated lipoprotein(a) (Lp[a]) is independently associated with atherosclerotic cardiovascular disease (ASCVD) risk, although this association may be modified by factors such as diabetes or hypertension.^1^^,^^2^ Current European and U.S. guidelines consider elevated Lp(a) a risk-enhancing factor and recommend at least 1 lifetime measurement, particularly when ASCVD risk stratification is uncertain.^1^^,^^2^ Carotid ultrasonography is a well-validated noninvasive method for detecting subclinical atherosclerosis and improving risk discrimination.^3^ This study investigated whether elevated Lp(a) is associated with carotid atherosclerosis and whether this relationship varies according to baseline ASCVD risk or diabetes status.

Apparently healthy middle-aged (40-69 years) participants from the general population in Greece were invited to participate on a voluntary basis in screening programs in 3 municipalities in Attica, Greece, during the period 2023 to 2025.^4^ Triplicate office blood pressure (BP) measurements were taken in a single study visit after 5 minutes sitting at rest using a validated automated upper-arm cuff BP monitor (Microlife BP Ηome). Results of recent fasting blood tests were recorded or performed if not available in the context of a simultaneous national ASCVD prevention screening program. Lp(a) measurements, reported in mg/dL, were obtained from private and public laboratories participating in the national screening program. Participants with established ASCVD were excluded. For ASCVD risk stratification (low-moderate, high, or very-high risk) age-dependent Systematic Coronary Risk Evaluation 2 (SCORE2) thresholds were used as follows: <2.5%, 2.5 to < 7.5%, and ≥7.5% for age <50 years, and <5%, 5 to < 10%, and ≥10% for age 50 to 69 years.^4^ SCORE2-Diabetes was used for individuals with diabetes. Carotid ultrasonography was performed by 2 trained clinicians using a portable device (Lumify Philips Healthcare), equipped with a linear array probe (L12-4) and connected (USB-C) to a tablet (Samsung Galaxy S8 Ultra 14.6”).^4^ Carotid plaque was defined as a focal structure protruding into the arterial lumen of at least 0.5 mm, or 50% of the surrounding intima-media thickness value, or demonstrated a thickness >1.5 mm (from the media-adventitia to the intima-lumen interface). In the presence of any plaque in the common or internal carotid artery, or carotid bifurcation bilaterally, carotid plaque score (CPS) was calculated by summing the maximum height of each plaque.^4^

Logistic regression models estimated carotid plaque probability using SCORE2 alone and SCORE2 plus Lp(a). Model discrimination for the predicted probabilities was assessed using area under the receiver operating characteristic curves (AUC) with 95% CIs. Logistic regression for carotid plaque presence was also performed with elevated Lp(a) (≥30 mg/dL) and ASCVD risk categories as predictors and assessing for interaction between Lp(a) and ASCVD risk category. Multivariable general linear models assessed the association of elevated Lp(a) and ASCVD risk category with CPS in individuals with carotid atherosclerosis, adjusting for diabetes, lipid-lowering, and antihypertensive drug treatment. Interaction terms assessed effect modification by ASCVD risk category and diabetes. Analyses were performed using IBM SPSS Statistics (version 30.0; IBM Corp.). The study was approved by the Ethics Committee of Sotiria Hospital and all participants provided signed informed consent.

A total of 963 individuals with complete data were analyzed (mean age 57.2 ± 8.0 years, male 43.1%, body mass index 27.6 ± 4.7 kg/m^2^, smokers 27.7%, diabetes 7.2%, antihypertensive/lipid-lowering drug treatment 41.6%/46.6%, respectively, systolic/diastolic BP 123.8 ± 14.9/76.8 ± 9.6 mmHg, SCORE2 5.2% ± 3.4%, 24.3% with Lp(a) ≥ 30 mg/dL). Participants were classified as low-moderate (n = 491, 51.0%), high (n = 416, 43.2%), and very-high (n = 56, 5.8%) ASCVD risk, according to SCORE2.

Receiver operating characteristic analysis demonstrated satisfactory discrimination of SCORE2 for detecting carotid atherosclerosis (AUC: 0.744; 95% CI: 0.713-0.775), whereas adding Lp(a) did not result in a significant improvement (AUC: 0.745; 95% CI: 0.714-0.776). The prevalence of carotid atherosclerosis differed significantly across low-moderate (37.7%), high (67.1%), and very-high (78.6%) ASCVD risk categories (*P* < 0.001), whereas no independent significant effect was revealed for elevated Lp(a) or its interaction with ASCVD risk category (Figure 1A). In individuals with carotid atherosclerosis, multivariable general linear model showed that elevated Lp(a) (β = 8.13; 95% CI: 4.91-11.34; *P* < 0.001) and ASCVD risk category (very high vs low-moderate risk: β = 5.00; 95% CI: 3.62-6.38; *P* < 0.001) were independently associated with higher CPS, after adjusting for diabetes, lipid-lowering, and antihypertensive drug treatment (Figure 1B). However, a significant interaction was observed between Lp(a) and ASCVD risk category (*P* < 0.001), indicating heterogeneity of the association between Lp(a) and plaque burden across ASCVD risk strata, with most pronounced differences among those at very-high ASCVD risk. No significant interaction was observed between Lp(a) and diabetes (*P* = 0.065). Sensitivity analyses using the guideline-endorsed Lp(a) threshold of ≥50 mg/dL yielded directionally similar findings. Elevated Lp(a) remained independently associated with higher CPS (β = 5.45; 95% CI: 1.56-9.33; *P* = 0.003), whereas the interaction between Lp(a) and ASCVD risk category was not significant (*P* = 0.059).

**Figure 1:**
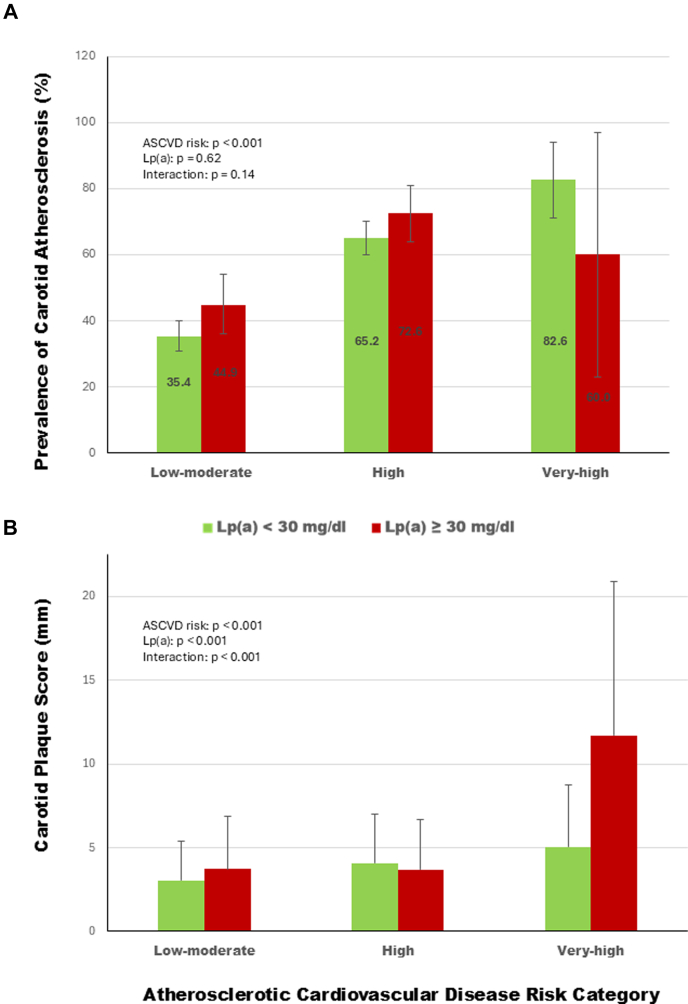
Prevalence and Burden of Carotid Atherosclerosis Across ASCVD Risk Categories (A) Prevalence and (B) burden of carotid atherosclerosis across atherosclerotic cardiovascular disease (ASCVD) risk categories stratified by lipoprotein(a) (Lp[a]) <30 vs ≥30 mg/dL. Error bars in A represent 95% CIs, whereas in B represent SD. Text boxes display *P* values for the main effects of the ASCVD risk category, elevated Lp(a), and their interaction (A, logistic regression model; B, multivariable general linear model).

In this community-based screening program in middle-aged adults without established ASCVD, elevated Lp(a) was not independently associated with the presence of carotid atherosclerosis after adjustment for ASCVD risk category. Moreover, Lp(a) addition to SCORE2 did not improve the detection of carotid atherosclerosis. These findings are in line with outcome studies showing that although Lp(a) is independently associated with ASCVD, its addition to contemporary risk equations results in modest, if any, improvement in overall model discrimination, with gains largely limited to risk reclassification in selected subgroups.^5^

However, in individuals with carotid atherosclerosis, elevated Lp(a) was associated with a substantially greater atherosclerotic burden, particularly at very-high ASCVD risk. These findings could suggest that Lp(a) acts primarily as a modifier of atherosclerotic disease severity rather than as a standalone determinant of atherosclerosis or a homogeneous risk enhancer across all risk categories. The strong association between Lp(a) and plaque burden aligns with the known proatherogenic, proinflammatory, and antifibrinolytic effects of Lp(a) that may accelerate plaque progression rather than just trigger its initial development.^1^^,^^2^

Limitations of this study include the cross-sectional design precluding causal inference, the voluntary participation in the screening program raising the possibility of selection bias toward more health-conscious individuals, and the limited generalizability to other age groups/populations. In addition, Lp(a) values were obtained from different laboratories and reported in mg/dL, which may introduce assay-related variability. The Lp(a) threshold (30 mg/dL) was selected based on prior epidemiological studies and to ensure adequate power for interaction analyses. Sensitivity analyses using the clinically endorsed Lp(a) threshold (50 mg/dL) demonstrated generally consistent findings, although interaction estimates were attenuated, likely due to reduced statistical power (smaller subgroup size). Lastly, the imaging methodology primarily focused on plaque presence and burden without detailed characterization of plaque phenotype, composition, or vulnerability.

In conclusion, elevated Lp(a) did not predict the mere presence of carotid atherosclerosis, but was associated with greater atherosclerotic burden among individuals with subclinical atherosclerosis, particularly at higher ASCVD risk. Although the addition of Lp(a) to SCORE2 did not meaningfully improve discrimination performance, the present findings support the emerging concept that elevated Lp(a) may be more consistently associated with cumulative atherosclerotic plaque burden than with plaque presence or short-term ASCVD risk discrimination. These findings should be considered exploratory and hypothesis-generating rather than directly practice-changing. Future longitudinal studies are needed to determine whether integrating Lp(a) measurement with imaging-based assessment of subclinical atherosclerosis may improve ASCVD risk refinement and preventive strategies.

## Funding support and author disclosures

Drs Elpen, Krka, Rafarm, Uni-Pharma, Velka, Vianex, and Viatris provided research grants for this study through the Special Account for Research Grants, 10.13039/501100005187National and Kapodistrian University of Athens. Dr Kollias received lecture/consulting fees by Astra-Zeneca, Boehringer In, Elpen, Menarini, 10.13039/100004319Pfizer, Rafarm, Servier, Uni-Pharma, Vianex, Viatris, and Winmedica. Dr Stergiou received lecture fees by AstraZeneca, Menarini, Servier, Viatris, and WinMedica; consulting fees by AstraZeneca, Lavipharm, Menarini, Sanofi-Aventis, and Viatris; and research grants by AstraZeneca, Menarini, Servier, and WinMedica. All other authors have reported that they have no relationships relevant to the contents of this paper to disclose.
